# Use of a Smartwatch for Assessment of the QT Interval in Outpatients with Coronavirus Disease 2019

**DOI:** 10.19102/icrm.2020.1100904

**Published:** 2020-09-15

**Authors:** Jason S. Chinitz, Rajat Goyal, Donna Chelle Morales, Melissa Harding, Samy Selim, Laurence M. Epstein

**Affiliations:** ^1^Southside Hospital, Northwell Health Physician Partners, Zucker School of Medicine at Hofstra/Northwell, Bay Shore, NY, USA

**Keywords:** Coronavirus, COVID-19, smartwatch, QT prolongation, wearable cardiac monitor

## Abstract

The coronavirus disease 2019 (COVID-19) pandemic has necessitated rapid implementation of innovative strategies to manage patients remotely to help reduce the risk of community and nosocomial transmission. This case demonstrates the use of an Apple Watch (Apple, Cupertino, CA, USA) to monitor for arrhythmias and QT prolongation in a patient with COVID-19 during home isolation.

## Introduction

During the ongoing coronavirus disease 2019 (COVID-19) pandemic, limitations in health care resources and the introduction of extensive protections against viral dissemination have forced the development and adoption of novel approaches to support previously routine patient management. Patients with COVID-19 infection are at risk for various cardiovascular and arrhythmic complications.^1^ In addition, medications being explored to treat COVID-19 infection, such as chloroquine, hydroxychloroquine, and azithromycin, carry a risk of cardiac complications, particularly a dose-dependent risk of QT prolongation and ventricular arrhythmia.^2,3^ In hospitalized patients, QT monitoring during the course of treatment with these medications may be performed using serial electrocardiograms (ECGs), telemetry, or even ambulatory telemetry monitors.^4^ However, the limited availability of such resources due to high demand alongside the precautions set in place to protect health care providers from contact with contagious patients has precluded standard monitoring techniques. Furthermore, most patients with COVID-19 infection are treated as outpatients, employing self-isolation to reduce the risk of nosocomial and community spread.^5^ A sudden and dramatic increased reliance on telehealth and remote ECG monitoring to manage these patients at home has therefore emerged.

Wearable and “smart” cardiac monitoring devices such as the Apple Watch series 4 (Apple, Cupertino, CA, USA) and the AliveCor Kardia system (AliveCor Inc., San Francisco, CA, USA) have the ability to record a rhythm strip essentially equivalent to lead I on a standard ECG and deliver the results via electronic transmission. While these devices have been validated for their ability to monitor heart rates and differentiate sinus rhythm from atrial fibrillation,^6^ their accuracy for other ECG-interpretation purposes has not been well studied to date. However, the waveforms produced have been shown to correlate reasonably well with standard ECG intervals including the corrected QT (QTc) interval^7^ and have previously been used to detect drug-induced QT interval prolongation.^8^

As patients with COVID-19 infection continue to overwhelm our global health care system and resources, the ability to remotely monitor patients for cardiac complications of COVID-19 infection and ensure their safety during pharmacologic treatment has become increasingly important. Currently available wearable cardiac monitoring devices may therefore play an important role in the management of patients in this pandemic.

We report herein a case of a patient with COVID-19 infection who was treated with a QT-prolonging medication and was able to be managed remotely with the assistance of a smartwatch device.

## Case presentation

**Figure 1:**
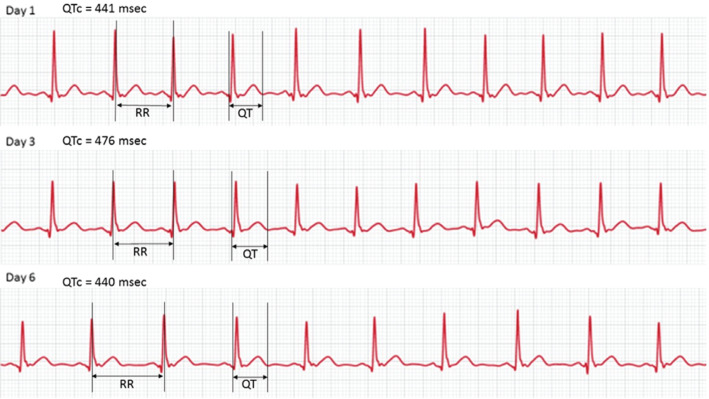
Rhythm strips used for QTc measurement. These rhythm strips were obtained from the patient’s Apple Watch during the course of treatment. The QT and RR intervals are measured as shown and the QTc interval was calculated using Bazett’s formula.

**Figure 2:**
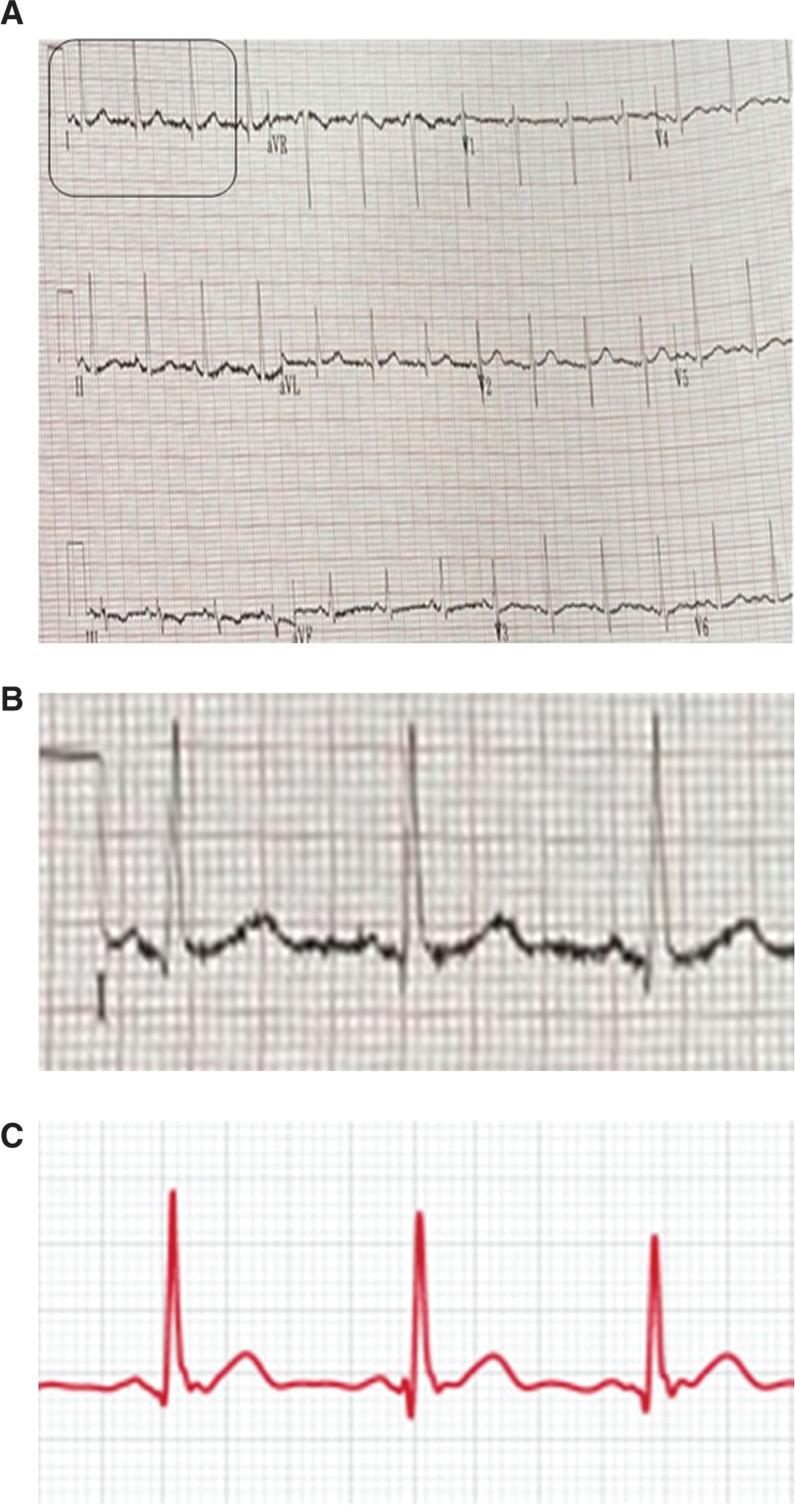
**A:** ECG obtained following the completion of treatment under home isolation. Note the consistency in the QT interval when measured in lead I or lead II (QT interval: 380 ms, RR interval: 690 ms, and QTc interval: 457 ms in both leads). **B:** Enhancement of lead I from the 12-lead ECG. **C:** Corresponding rhythm strip obtained from the Apple Watch after treatment, where a very similar waveform morphology and QT measurements as compared with lead I on the 12-lead ECG can be noted.

A 40-year-old female physician with no prior cardiac history presented with fevers, chills, cough, and dyspnea. Due to known exposures to patients and close relatives with confirmed COVID-19 infection, she was immediately placed in home isolation and managed by her primary care provider and cardiologist using telemedicine. Due to persistent fevers, she was prescribed hydroxychloroquine (400 mg twice daily for one day and then 200 mg twice daily for four days to complete a five-day course). No baseline ECG was available; however, she was considered to be at moderate risk for drug-associated QT prolongation due to a Tisdale risk score of seven points.^9^ As such, she used her Apple Watch to record rhythm strips approximately two to three hours after each dose of hydroxychloroquine administration and transmitted these results to her cardiologist **(Figure 1)**. Her QTc interval was 441 ms at baseline (measured using Bazett’s correction), increased to 476 ms after the third dose, and then returned to baseline at 440 ms one day after the completion of the five-day course. No arrhythmias were detected during the course of treatment by the Apple Watch. She was able to complete treatment at home and, as her symptoms improved, a 12-lead ECG was subsequently performed in the hospital **(Figure 2)**, which confirmed the waveform measurements obtained by the Apple Watch (QTc interval was 457 ms on ECG) and demonstrated consistency between limb lead measurements (QT interval was 380 ms in both leads I and II).

## Discussion

Severe acute respiratory syndrome coronavirus 2 (SARS-CoV-2), the virus behind COVID-19, has a high rate of transmission in the community as well as within health care facilities, forcing a new emphasis to be placed on the use of telemedicine and remote monitoring. While telehealth has previously been effective in managing patients, in this context, performing necessary prior steps, including ECGs and the application of mobile cardiac telemetry monitors, is precluded. At the same time, findings of cardiac complications related to SARS-CoV-2 infection and the potential cardiac toxicity associated with common pharmacologic treatments have prompted an increased need for easily accessible cardiac monitoring modalities that do not rely on physical contact with or the patient’s presentation to a health care facility. Remote patient management using smart device–based electrocardiography is therefore a valuable alternative to previously standard monitoring modalities.

The waveforms provided by smart device–based ECGs have not been vigorously assessed for the purpose of QT measurement and no outcomes studies based on this strategy have been conducted to date. The rhythm strip obtained by such devices is typically derived from the same vector as lead I of a standard 12-lead ECG; the waveforms are overlaid on standard ECG grids with recordings at 25 mm/s and 10 mm/mV, allowing QT and RR interval measurements to be determined in milliseconds. The QTc interval can therefore be derived in the same manner as that adopted when using a standard 12-lead ECG. A recent validation study performed in 129 healthy volunteers showed a reasonable concordance between QTc intervals measured from the Apple Watch and a 12-lead ECG (mean difference: −11.67 ± 8.32 ms; r = 0.57).^7^ In addition, a prior study demonstrated the ability to use a smartphone-based heart monitor to accurately detect QTc prolongation in a patient receiving QT-prolonging medications, the results of which revealed a difference of 4 ms with standard deviation of 11 ms as compared with the 12-lead ECG.^8^ Elsewhere, another study found that QTc measurement using a single-lead handheld mobile device is feasible, but the QTc measurement was underestimated (23 ms; 95% confidence interval: 13–34 ms) when only a lead I rhythm strip was obtained; when a vector analogous to lead II could be recorded, QTc measurements had a very close correlation with the maximum QTc interval determined by 12-lead ECG.^10^ Importantly, the change in QTc during treatment with QT-prolonging medications has also been correlated with an increased risk of torsades de pointes,^11^ indicating that serial measurements collected using a single lead (such as when using a smart device) may provide additional value during risk stratification even when the measured QTc value is imprecise.

Further validation and optimization of recording techniques are required before this technology can be promoted for routine assessment of the QT interval. If this approach is adopted, QTc measurement can likely be automated to improve the ease of measurement and reduce measurement errors. The lack of multiple contiguous leads obtainable by most wearable devices limits the sensitivity of these modalities for the detection of QT abnormalities, which may be more pronounced in some leads than others. While lead II is most commonly used for QT measurement, the optimal lead for QT measurement does vary and the use of the same lead for serial assessments is important to accurately interpret QT changes while on medication. Lead I is frequently adequate for interpretation and the QTc measurement from lead I has been shown to differ by an average of 7.5 ms from that obtained from lead II.^12^ Various ECG vectors, including lead II, can be obtained by both the Apple Watch and Kardia Pro devices by repositioning the device on the patient’s body.^13^ Accordingly, single-lead mobile cardiac outpatient telemetry units have previously been approved by the United States Federal Drug Administration for QTc measurement. Although the quality of the electrograms produced by smart devices may be affected by a high level of skin-to-electrode impedance, it remains generally consistent relative to other monitoring technologies and has been well-validated for use in rhythm interpretation.

## Conclusion

Despite the above limitations, the COVID-19 pandemic has required providers to move swiftly to use all current resources at their disposal to both manage patients safely and minimize the spread of infection, particularly in the health care environment. Smart monitoring devices such as the Apple Watch Series 4 permits monitoring of the heart’s rhythm, rate, and QT interval and thereby can provide a distinct advantage in the management of patients with SARS-CoV-2 infection during home isolation. This case demonstrates one novel approach to patient management under these difficult circumstances and could be considered further as a means to limit the risk of transmission in future outbreaks.
